# Supplementary material for: Innovative deep matching algorithm for stock portfolio selection using deep stock profiles
Source: PLoS One. 2020 Nov 4;15(11):e0241573. doi: 10.1371/journal.pone.0241573 (PMC7641377; doi:10.1371/journal.pone.0241573)
Supplement: S1 File — (PDF) [file pone.0241573.s001.pdf]

# Chinese text sentiment classification model in financial field

Recently, textual representation and classification based on deep learning has become a research hotspot, achieving excellent performances on several open-source datasets [1]. However, textual representation and classification in specific languages and domains remains a challenge [2]. To more accurately extract the sentiment features of stock profiles from financial social media texts, we customized the methods of word segmentation and labeling training data for financial Chinese texts. These methods were used to train a word vector model and a text sentiment classification model based on deep learning.

- **Text sentiment labeling method**

  We divided the social media texts of stock topics into three types of sentiment orientations: positive, neutral, and negative. Positive sentiment includes bullishness, elation, excitement, surprise, and optimism, probably indicating an investor's attitude of intending to buy stocks. Neutral sentiment, such as a vague expression of opinion or confusion, probably indicates that investors hold a wait-and-see attitude toward a stock or intend to hold it for the time being. Negative sentiment, such as bearishness, doubt, frustration, fear, sadness, and anger, probably indicates that investors intend to sell stocks. The digits 1, 0, and -1 were used to represent positive, neutral, and negative investor sentiments, respectively. We used three professional annotators who had a rich theoretical knowledge of securities investment. Each text was labeled by the three annotators, and the labeled result was based on the "best-of-three sets" rule. One hundred texts were randomly selected per 10,000 annotation results for verification. If the accuracy was below 95%, the texts would be re-labeled. For texts that were too short or contained misspellings, their sentiment orientations were labeled as neutral. A total of 120,000 texts were selected randomly from 30 million texts on stock topics in social media and were labeled. Out of these, 42,068, 59,095, and 18,837 texts were labeled as positive, neutral, and negative, respectively.

- **Chinese text segmentation system in financial field**

  Chinese text needs to be segmented according to semantics, and the current Chinese word segmentation systems are not aimed at the financial field. Therefore, to train a superior text representation model, it is first necessary to improve the accuracy of the word segmentation system. Based on the discussion corpus of financial social media, we generated a professional word segmentation dictionary using manual extraction by experts. The dictionary included stock names, network terms (ghost stocks, penny stocks, junk stocks, etc.), jargon, vernacular terms, social media-specific emojis, main business of enterprises, financial report terms, etc. Based on this custom dictionary, the Ansj word segmentation tool was used to build a Chinese text segmentation system. Ansj was improved from the Chinese Academy of Sciences' Ictclas [3], which was a Java implementation of Chinese word segmentation based on n-grams, conditional random fields, and the hidden Markov model [4]. A total of 13,945 words in a custom dictionary were added into the Ansj default general dictionary to perform Chinese text segmentation of stock topic discussions.

- **Text representation and classification models based on deep learning**

  The word2vec algorithm [5] was used to train our text representation model. A total of 83,979,599 posts and comments relating to 1000 stocks, most of which were under 100 words, were used as a training corpus. If the i-th word in a given sentence corresponded to a k-dimensional vector $x_i \in O^k$, the sentence could be formulated as $s_{1:m} = x_1 \oplus x_2 \oplus ... \oplus x_m$, where $\oplus$ denotes the concatenation operator; m represents the number of words contained in the longest sentences in the corpus after word segmentation, and $s_{1:m}^{(j)} \in O^{m \times k}$ indicates the representation matrix of the j-th sentence in the corpus. For sentences less than m in length after word segmentation, we filled in the difference with zero vectors. We adopted a deep CNN algorithm [6] to train the text sentiment classification model. The output dimensionality of word vectors was 128, the minimum number of omits was 10, and the window was 3 in the pre-trained word2vec model. The filter sizes of the CNN model were 3, 4, and 5; the number of filters was 128; the dropout was 0.5; the iterations were 200, and the batch size was 64. Under the method of cross validation, the 120,000 annotated data were divided into training, testing, and verification sets, with a model accuracy of 73% on the testing set.

# Deep text matching algorithms

- **DRMM**

  Deep relevance matching model (DRMM) [7] in information retrieval field refers to the matching between queries and documents, which is to be distinguished from traditional textual semantic matching between two sentences. As shown in Fig 1, DRMM represents each word in the queries and documents with the pre-trained word vectors.

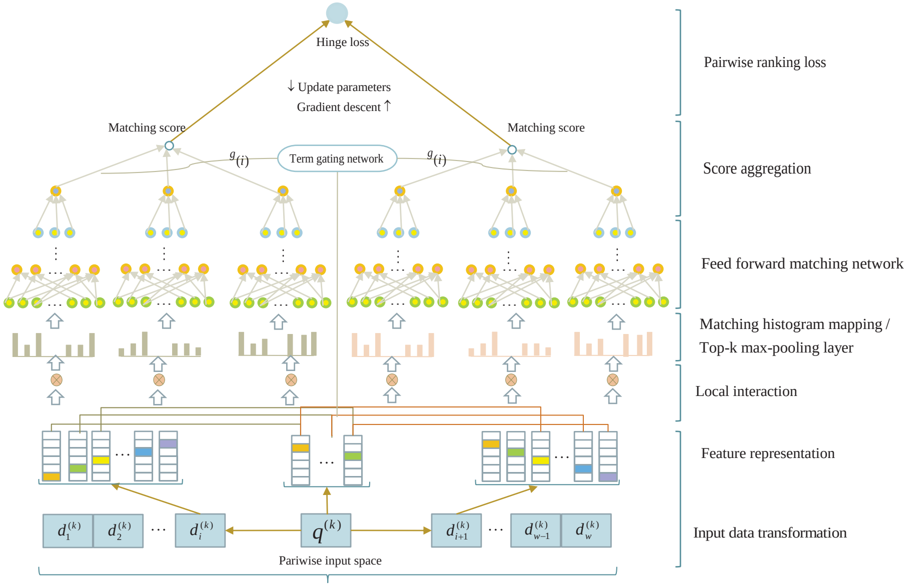


**Fig 1. DRMM architecture.** As it is shown the feature representation layer corresponds to weekly stock feature matrices and weekly stock selection target matrices. The middle local interaction, matching histogram mapping, feed forward matching network, and score aggregation layers are uniquely designed in this model.

DRMM calculates cosine similarities between each word in the queries and all

words in corresponding documents, and grades the similarities of each query-document pair for obtaining the matching histograms with fixed length (Eq (1)). The matching histograms then are input into a feed forward matching network to extract higher level similarity information (Eq (2)). After obtaining the weight distributions of different query words (Eq (3)), the weighted sum of the correlation scores of query-document pairs are calculated to obtain the final correlation scores (Eq (4)). In the model training, DRMM adopts hinge loss function to compute the pairwise ranking loss (Eq (5)).

$$z_i^{(0)} = h(w_i^q \otimes d), i = 1, ..., M \tag{1}$$

$$z_i^{(l)} = tanh(W^{(l)} z_i^{l-1} + b^{(l)}), i = 1, ..., M, l = 1, ..., L \tag{2}$$

$$g_i = \frac{exp(w_g x_i^q)}{\sum_{j=1}^{M} exp(w_g x_j^q)}, i = 1, ..., M \tag{3}$$

$$s = \sum_{i=1}^{M} g_i z_i^{(L)} \tag{4}$$

$$\varsigma(q, d^+, d^-; \Theta) = max(0, 1 - s(q, d^+) + s(q, d^-) \tag{5}$$

where $\otimes$ denotes the interaction operator (i.e., the matching function of each pair of word vectors) and $h(\cdot)$ indicates the mapping function from similarities to histogram features with fixed length. $z_i^{(0)}$ denotes the intermediate hidden layers for the i-th query term and $g_i$ denotes the aggregation weight that is produced by term gating network. $W^{(l)}$ and $b^{(l)}$ denote the l-th weight matrix and the l-th bias term, respectively, and the two are shared across different query terms. $x_i^q$ indicates the vector representation of each word in the queries. $w_g$ is a weight vector training parameter and shares the same dimensionality with $x_i^q$. $d^+$ is a more relevant document than $d^-$ for the query $q$.

- **Conv-KNRM**

  Kernel-based neural ranking model (K-NRM) [8] first performs the vector representation for each word in the query item with $h_q$ words and in the document item with $h_d$ words and calculates the cosine similarities between each word in the query item and all words in the document item in turn, thus obtaining a similarity matrix $M^{h_q,h_d} = \begin{bmatrix} \vec{v_1} \\ ... \\ \vec{v_m} \end{bmatrix}$. Then a gaussian kernel transformation is performed for each row in $M^{h_q,h_d}$ and corresponding vectors $M_{ij}^{h_q,h_d}$ are obtained. The elements in vectors represented different kernel transformation values (Eq (7)–(10)).

$$\vec{g_i^h} = relu(W^h \cdot T_{i:i+h} + b^h) M_{ij}^{h_q,h_d} = cos(g_i^{h_q}, g_i^{h_d}), \tag{6}$$

$$K_k(M_i) = \sum_j exp(-\frac{(M_{ij} - \mu_k)^2}{2\sigma_k^2}), \tag{7}$$

$$\vec{K}(M_i) = K_1(M_i), ..., K_k(M_i), \tag{8}$$

$$\phi(M) = \sum_{i=1}^{n} log \vec{K}(M_i), \tag{9}$$

$$f(q, d) = tanh(w^T \phi(M) + b) \tag{10}$$

Where $\vec{g_i^h}$ indicates the embedding of the i-th h-gram, and the f-th element in $g_i^h$ is the score of the f-th filter. When adopting a pairwise method, the loss function

is formulated as Eq (11):

$$\zeta(w,b,V) = \sum_{q} \sum_{d^+,d^- \in D_q^{+,-}} max(0, 1 - f(q,d^+) + f(q,d^-)) \quad (11)$$

Where relevant $d^+$ ranks higher than the irrelevant $d^-$, and learning parameters include $w, b$, and $V$. After logarithm fetch on kernel transformation values, which are added up to obtain Soft-TF characteristics. These characteristics are inputted into the full connection layer to obtain the final classification Eq (10). Compared with K-NRM, convolution kernel-based neural ranking model (Conv-KNRM) [9] adds n-gram convolutions Eq (6) and the number of layers in primitive mode to capture more subtle semantic entities and fine intersection granularity (Fig 2).

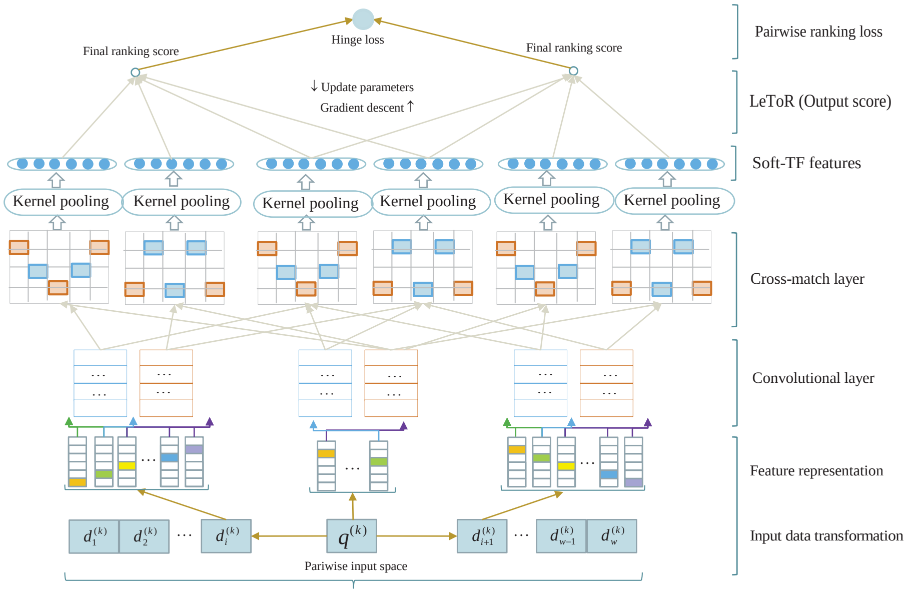


**Fig 2. Conv-KNRM architecture.** As it is shown the feature representation layer corresponds to weekly stock feature matrices and weekly stock selection target matrices. The middle convolutional, cross-match, soft-TF features, and LeToR layers are uniquely designed in this model.

- **MV-LSTM**

  Multi-view LSTM ( MV-LSTM) is a deep neural network architecture for semantic matching with multiple positional sentence representations, which is designed for more easily capturing the local context information in the process of text matching [10]. MV-LSTM uses a bidirectional long short-term memory algorithm (Bi-LSTMs) [11] to generate multi-position sentence representations (Fig 3).

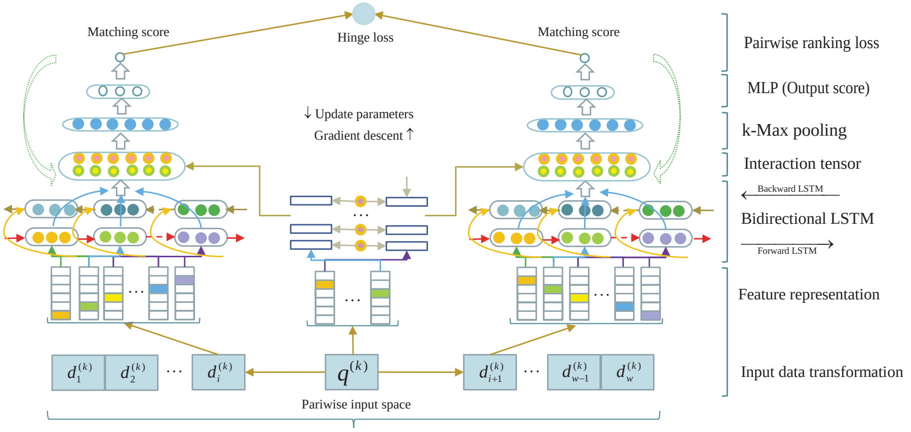


**Fig 3. MV-LSTM architecture.** As it is shown the feature representation layer corresponds to weekly stock feature matrices and weekly stock selection target matrices. The middle bidirectional LSTM, interaction tensor, k-Max pooling, and MLP layers are uniquely designed in this model.

Bi-LSTMs can capture the backward and forward directional information of sequence, with the outpt at each position being the connection of two vectors $\overrightarrow{h_t}$ and $\overleftarrow{h_t}$ in both directions. If defining one sentence $T = \{t_{(1)}, t_{(2)}, \cdots, t_{(k)}, \cdots, t_{(w)}\}$, $t_{(k)}$ as the input of the algorithm, $t_{(k)}$ represents the vector representation of a word at position t, and a representation $h_{(t)}$ at position t is outputted after being calculated with Eq (12)–(16).

$$i_t = \sigma(W_i x(t) + U_i h(t-1) + b_i), \quad (12)$$
$$f_t = \sigma(W_f x(t) + U_f h(t-1) + b_f), \quad (13)$$
$$C_t = f_t C_{t-1} + i_t \tanh(W_c x(t) + U_c h(t-1) + b_c), \quad (14)$$
$$o_t = \sigma(W_o x(t) + U_o h(t-1) + b_o), \quad (15)$$
$$h_t = o_t \tanh(C_t), \quad (16)$$

Where $i_t$, $f_t$, and $o_t$ represent the input, forget, and output gates, respectively. Vector $C_t$ is a memory unit used to store information. $h_t$ denote a sigmoid function. $W$, $U$, or $b$ are matrices including network structure parameters. The sentence vector representation at the t-th position $p_t = [\overrightarrow{h_t}^T, \overrightarrow{h_t}^T]^T$ is obtained by connecting $\overrightarrow{h_t}$ and $\overleftarrow{h_t}$, where $(\cdot)^T$ represents the performing of substitution operation. Based on sentence vector representations, MV-LSTM applies a similarity calculation formula with parameters (Eq (17)) to compute the interaction between $p_{X_i}$ and $p_{Y_i}$, where $p_{X_i}$ and $p_{Y_i}$ are the representations of sentence $S_X$ at the i-th position and sentence $S_Y$ at the j-th position, respectively. The Eq (17) will output an interaction tensor , which is more capable of mapping the diversity of language representations. Then, k-max pooling layer extracts the top k strongest local information from similarity matrix or tensor according to Eq (19), and generates a higher-level vector representation (Eq (17)) through the fully connected hidden layer. Multi-layer perceptron [12] calculates the matching

scores with Eq (20). The loss function of MV-LSTM is formulated as Eq (21).

$$s(u,v) = f(u^T M^{1:c} v + W_{uv} \begin{bmatrix} u \\ v \end{bmatrix} + b), \tag{17}$$

$$C^k_{max} = \max[C_{j,l}], \tag{18}$$

$$r = f(W_r C^k_{max} + b_r), \tag{19}$$

$$s = W_s r + b_s, \tag{20}$$

$$\zeta(S_X, S^+_Y, S^-_Y) = max(0, 1 - s(S_X, S^+_Y) + s(S_X, S^-_Y), \tag{21}$$

where $M^i, i \in [1, \ldots, c]$ is one slice of the tensor parameters, $W_{uv}$ and b are parameters of the linear part. f is a non-linear function ($f(z) = max(0, z)$). $W_r$ and$W_s$ stand for the parameter matrices, and $b_r$ and $b_s$ are corresponding biases. $S^+_Y$ and $S^-_Y$ represent positive and negative samples, respectively.

## References


1. Van der Maaten L, Hinton G. Visualizing data using t-SNE. J Mach Learn Res. 2008; 9:2579-2605.

2. Partridge M, Calvo RA. Fast dimensionality reduction and simple PCA. Intell Data Anal. 1998; 2(3):203-214.

3. Zhang H-P, Yu H-K, Xiong D-Y, Liu Q. HHMM-based Chinese lexical analyzer ICTCLAS. In: SIGHAN '03: Proceedings of the second SIGHAN workshop on Chinese language processing-Volume 17, Sapporo Japan, Association for Computational Linguistics, 2003;184-187.

4. Fine S, Singer Y, Tishby N. The hierarchical hidden Markov model: analysis and applications. Mach Learn. 1998; 32(1):41-62.

5. Mikolov T, Sutskever I, Chen K, Corrado G, Dean J. Distributed representations of words and phrases and their compositionality. In: Advances in Neural Information Processing Systems 26 (NIPS 2013). Lake Tahoe, Nevada, USA; 2013. p. 3111-3119.

6. Kim Y. Convolutional neural networks for sentence classification. In: Proceedings of the 2014 Conference on Empirical Methods in Natural Language Processing (EMNLP). Doha, Qatar; 2014. p. 1746–1751.

7. Guo J, Fan Y, Ai Q, Croft WB. A deep relevance matching model for ad-hoc retrieval. In: CIKM '16: Proceedings of the 25th ACM International on Conference on Information and Knowledge Management. Indianapolis, Indiana, USA; 2016. p. 55-64.

8. Xiong C, Dai Z, Callan J, Liu Z, Power R. End-to-end neural ad-hoc ranking with kernel pooling. In: SIGIR '17: Proceedings of the 40th International ACM SIGIR Conference on Research and Development in Information Retrieval. Shinjuku Tokyo, Japan; 2017. p. 55-64.

9. Dai Z, Xiong C, Callan J, Liu Z. Convolutional neural networks for soft-matching n-grams in ad-hoc search. In: WSDM '18: Proceedings of the Eleventh ACM International Conference on Web Search and Data Mining. Los Angeles, California, USA; 2018. p. 126-134.

10. Wan S, Lan Y, Guo J, Xu J, Pang L, Cheng X. A deep architecture for semantic matching with multiple positional sentence representation. In: Thirtieth AAAI Conference on Artificial Intelligence (AAAI-16). Phoenix, Arizona, USA; 2016.

11. Graves A, Schmidhuber J. Framewise phoneme classification with bidirectional LSTM and other neural network architectures. Neural Netw. 2005; 18(5-6):602-610.

12. Koskela T, Lehtokangas M, Saarinen J, Kaski K. Time series prediction with multilayer perceptron, FIR and Elman neural networks. In: Proceedings of the World Congress on Neural Networks. San Diego, USA; 1996. p. 491-496.
