# Supplementary material for: Innovative deep matching algorithm for stock portfolio selection using deep stock profiles
Source: PLoS One. 2020 Nov 4;15(11):e0241573. doi: 10.1371/journal.pone.0241573 (PMC7641377; doi:10.1371/journal.pone.0241573)
Supplement: Attachment — Submitted filename: Response to Reviewers.doc [file pone.0241573.s016.doc]

**Point-by-point response to comments of Reviewers**

Comments to the Author

1. Is the manuscript technically sound, and do the data support the conclusions?

The manuscript must describe a technically sound piece of scientific research with data that supports the conclusions. Experiments must have been conducted rigorously, with appropriate controls, replication, and sample sizes. The conclusions must be drawn appropriately based on the data presented.

Reviewer #1: Yes

Reviewer #2: Partly

________________________________________

2. Has the statistical analysis been performed appropriately and rigorously?

Reviewer #1: Yes

Reviewer #2: N/A

________________________________________

3. Have the authors made all data underlying the findings in their manuscript fully available?

The PLOS Data policy requires authors to make all data underlying the findings described in their manuscript fully available without restriction, with rare exception (please refer to the Data Availability Statement in the manuscript PDF file). The data should be provided as part of the manuscript or its supporting information, or deposited to a public repository. For example, in addition to summary statistics, the data points behind means, medians and variance measures should be available. If there are restrictions on publicly sharing data—e.g. participant privacy or use of data from a third party—those must be specified.

Reviewer #1: No

Reviewer #2: Yes

________________________________________

4. Is the manuscript presented in an intelligible fashion and written in standard English?

PLOS ONE does not copyedit accepted manuscripts, so the language in submitted articles must be clear, correct, and unambiguous. Any typographical or grammatical errors should be corrected at revision, so please note any specific errors here.

Reviewer #1: Yes

Reviewer #2: Yes

________________________________________

5. Review Comments to the Author

Please use the space provided to explain your answers to the questions above. You may also include additional comments for the author, including concerns about dual publication, research ethics, or publication ethics. (Please upload your review as an attachment if it exceeds 20,000 characters)

**Reviewer #1: Referee Report**

Journal: PLOS ONE

Title: Innovative deep matching algorithm for stock portfolio selection using deep stock profiles

Manuscript Number: PONE-D-20-15612

Date: 2020/06/18

Summary

This paper applies the innovative deep matching algorithm to select stock portfolio and test the portfolio strategies. Basically, the topic is interesting for readers interested in stock markets. It is commendable that the authors conduct a vast array of tasks to skillfully introduce their innovative deep matching algorithm, but I still have some reservation about the results of this study. Specific comments are outlined below and I hope my comments can be useful for the authors to enhance this paper.

Major Comments

1. The authors need to be clear on their contribution. I would expect to see a more comprehensive literature review that help readers understand the role of this article in the existing studies. For example, it is necessary to mention clearly what observations are the same or different compared with previous studies using similar approaches. When the observations are different, it is necessary to provide possible explanations for the difference. This could further enhance the contributions of this paper.

2. In methodology, professional technique for stock portfolio selection goes beyond traditional methods so as to be difficult for most readers in the financial field. I would like to recommend the authors to shorten the experimental procedure and lengthen the discussion on the results instead.

3. For empirical results, I suggest the authors to provide economic and financial implications. Providing results are not enough for stock portfolio selection. Readers would like to learn how such results can be applied to their trading strategies. This is an important issue that authors should improve. Please indicate implications and how the general results help stock traders to form investment strategies or conduct market arbitrage. It would make this paper a lot more interesting.

4. The conclusions repeat the operational approach. It would be better for the authors to provide some of concrete takeaways from this paper.

Minor Comments

1. Several figures are blurry.

2. What is the information ratio?

…

**Reviewer #2:** The document uses a mixture of different tools for natural language processing and information retrieval that are in standard use. Firstly, the document lacks the foundational literature of portfolio theory, as the title suggests that it addresses this problem, the authors suggest that it is based on the factor models developed by FAMA-French but this is not clear, the study bases its motivation on the challenges presented by financial time series regarding a low signal-to-noise ratio which are not well explained, in addition to not explaining from the concept and why it is a problem in finance, it does not explain how its proposed model attacks this problem, since in engineering it is not a minor problem and is addressed in fields such as signal processing in finance is also a relevant problem and deserves to be better explained and do not leave it to the reader. the acronym TS-Deep-LtM, is not clearly explained, Deep given the use of Deep Neural Networks, LtM for Learning to Rank, and TS for Time Series? that is not clear. Treating this topic of the document, there is no evidence on bias and variance assessing to see that the neural networks are not presenting overfitting, since in the financial time series the power of generalization is a key point for the success of a step to production, neither is it presents evidence on whether it was necessary to regularize the model or fine-tuning of the hyperparameters of the neural networks, there is no further evidence on the criteria for performing "feature selection" according to the author, since it is not performed under any objective criteria, the feature selection It is a process that can be done manually or automatically depending on a clear objective. The proposal is novel since it uses NLP methods to address the process of stock selection or stock picking, on this, it must be said that the title does not refer to the content because it is not addressed as a portfolio problem given that the found weightings by the proposed model are not presented and the selection of assets that it makes throughout the analysis sliding window from 2010 to 2017 also, is not presented. About the Data preprocessing could be better, or the paper could document reasons for these particular preprocessing choices. There are several writing sequences that have problems, for example with the use of subjects, plurals and singular in English.

**Reviewer #1**

Response: Thanks for the professional comments and suggestions. We have revised the manuscript and the changes made were highlighted in a marked-up copy of our manuscript in this way: added text is blue and set in sans-serif, and a red footnote is created for each discarded piece of text.

1. We have added the **discussion section in Page 15 line 584 to Page 16 line 662**. A comprehensive literature review is presented to help readers understand the role of this article in the existing studies and enhance the contributions of this paper.
2. We have shortened the experimental procedure, including the construction process of a text sentiment classification model **in Page 5 line 191 to Page 6 line 220** and the introduction of deep text matching methods **in Page 7 line 245 to Page 8 line 307**. We added a discussion section **in Page 15 line 584 to Page 16 line 662**.
3. In the result section, we added a application scheme of the experimental results in the quantitative investment (**Page 15 line 573 to 583**). In the intelligent system framework for stock selection, we modified the application module in Fig 16 (**Page 14 line 569 to Page 15 line 572**). In addition, in the methodology section (**Page 8 line 308 to Page 9 line 317**), we have revised the part of manuscript to present how to build a portfolio based on deep matching models. In the experiment design section (**Page 11 line 406 to 415**), we have revised the part of manuscript to present how to design a trading strategy based on a portfolio and to present the weight distribution of stocks and the selection of assets in a trading strategy. They can help readers to learn how such results can be applied to their trading strategies.
4. We have reorganized the conclusion section **in Page 16 line 663 to Page 17 line 672**.

Minor Comments

1. We have improved the resolution of figures while meeting the requirements of the journal. Please download the original Figures for viewing.
2. We have added the explanations for the information ratio and other evaluation metrics **in Page 12 line 434 to 470**.

**Reviewer #2**

Response: Thanks for the professional comments and suggestions. We have revised the manuscript and the changes made were highlighted in a marked-up copy of our manuscript in this way: added text is blue and set in sans-serif, and a red footnote is created for each discarded piece of text.

1. Firstly, the document lacks the foundational literature of portfolio theory, as the title suggests that it addresses this problem,

**Response**: We have added the foundational literature of portfolio theory in the introduction section (**Page 1 line 1 to Page 2 line 20**), which reviews the development of portfolio theory and its practical applications and helps to understand the relationship between the problem posed in this paper and the technical approach we adopted.

2. the authors suggest that it is based on the factor models developed by FAMA-French but this is not clear,

**Response**: We have revised the part of the manuscript about the factor models **in Page 3 line 71 to 77**, which clearly states the FAMA-French factor model as a basis of this study.

3. the study bases its motivation on the challenges presented by financial time series regarding a low signal-to-noise ratio which are not well explained, in addition to not explaining from the concept and why it is a problem in finance, it does not explain how its proposed model attacks this problem, since in engineering it is not a minor problem and is addressed in fields such as signal processing in finance is also a relevant problem and deserves to be better explained and do not leave it to the reader.

**Response**: We have revised the part of the manuscript to explain a low signal-to-noise ratio. The concept of signal-to-noise ratio (**Page 2 line 26 to 27**), why it is a problem in finance (**Page 2 line 27 to 31**), and how our proposed model attacks this problem are presented (**Page 2 line 56 to Page 3 line 65**).

4. the acronym TS-Deep-LtM, is not clearly explained, Deep given the use of Deep Neural Networks, LtM for Learning to Rank, and TS for Time Series? that is not clear.

**Response**: We have revised the part of the manuscript about acronym TS-Deep-LtM **in Page 4 line 118 to 119**, Deep given the use of deep neural networks, LtM for learning-to-match, and TS for Time Series.

5. Treating this topic of the document, there is no evidence on bias and variance assessing to see that the neural networks are not presenting overfitting, since in the financial time series the power of generalization is a key point for the success of a step to production,

**Response**: In the result section (**Page 14 line 535 to 540**), we added Figure 14 to evaluate the generalization ability of the deep matching models. The models are not presenting overfitting. We added reasons for the good generalization ability of the models in the discussion section (**Page 16 line 622 to 626**).

6. neither is it presents evidence on whether it was necessary to regularize the model or fine-tuning of the hyperparameters of the neural networks,

**Response**: It is necessary to fine-tuning of the hyper-parameters of the neural networks. In the methodology section (**Page 8 line 298 to 307**), we added the part of the manuscript about the hyper-parameters used in the model tuning phase and corresponding optional parameter values. In the result section (**Page 14 line 535 to 537**), we added a appendix file (S7 File) to present the optimal parameter combinations generated by the model tuning module during TS-Deep-LtM model training.

7. there is no further evidence on the criteria for performing "feature selection" according to the author, since it is not performed under any objective criteria, the feature selection It is a process that can be done manually or automatically depending on a clear objective.

**Response**: We have added the description about the criteria for performing "feature selection" **in Page 9 line 348 to 351**. The "feature selection" criteria for traditional factors is described in **Page 9 line 353 to Page 10 line 361**. The "feature selection" criteria for social media factors is described in **Page 10 line 363 to 374**.

8. The proposal is novel since it uses NLP methods to address the process of stock selection or stock picking, on this, it must be said that the title does not refer to the content because it is not addressed as a portfolio problem given that the found weightings by the proposed model are not presented and the selection of assets that it makes throughout the analysis sliding window from 2010 to 2017 also, is not presented.

**Response**: In the methodology section (**Page 8 line 308 to Page 9 line 317**), we have revised the part of manuscript to present how to build a portfolio based on deep matching models. In the experiment design section (**Page 11 line 406 to 415**), we have revised the part of manuscript to present how to design a trading strategy based on a portfolio and to present the weight distribution of stocks and the selection of assets in a trading strategy.

9. About the Data preprocessing could be better, or the paper could document reasons for these particular preprocessing choices.

**Response**: We have added the part of manuscript (**Page 10 line 391 to 393**) and Figure 7 to present our specific data preprocessing process in this study.

10. There are several writing sequences that have problems, for example with the use of subjects, plurals and singular in English.

**Response**: For some writing sequences that have problems, we have made corresponding modifications and they are highlighted in a marked-up copy of our manuscript in this way: added text is blue and set in sans-serif, and a red footnote is created for each discarded piece of text.
