# Supplementary material for: Innovative deep matching algorithm for stock portfolio selection using deep stock profiles
Source: PLoS One. 2020 Nov 4;15(11):e0241573. doi: 10.1371/journal.pone.0241573 (PMC7641377; doi:10.1371/journal.pone.0241573)
Supplement: Attachment — Submitted filename: Response to Reviewers.doc [file pone.0241573.s018.doc]

**Point-by-point response to comments of Reviewers**

Comments to the Author

1. If the authors have adequately addressed your comments raised in a previous round of review and you feel that this manuscript is now acceptable for publication, you may indicate that here to bypass the “Comments to the Author” section, enter your conflict of interest statement in the “Confidential to Editor” section, and submit your "Accept" recommendation.

Reviewer #1: (No Response)

Reviewer #3: All comments have been addressed

________________________________________

2. Is the manuscript technically sound, and do the data support the conclusions?

The manuscript must describe a technically sound piece of scientific research with data that supports the conclusions. Experiments must have been conducted rigorously, with appropriate controls, replication, and sample sizes. The conclusions must be drawn appropriately based on the data presented.

Reviewer #1: (No Response)

Reviewer #3: Yes

________________________________________

3. Has the statistical analysis been performed appropriately and rigorously?

Reviewer #1: (No Response)

Reviewer #3: N/A

________________________________________

4. Have the authors made all data underlying the findings in their manuscript fully available?

The PLOS Data policy requires authors to make all data underlying the findings described in their manuscript fully available without restriction, with rare exception (please refer to the Data Availability Statement in the manuscript PDF file). The data should be provided as part of the manuscript or its supporting information, or deposited to a public repository. For example, in addition to summary statistics, the data points behind means, medians and variance measures should be available. If there are restrictions on publicly sharing data—e.g. participant privacy or use of data from a third party—those must be specified.

Reviewer #1: (No Response)

Reviewer #3: Yes

________________________________________

5. Is the manuscript presented in an intelligible fashion and written in standard English?

PLOS ONE does not copyedit accepted manuscripts, so the language in submitted articles must be clear, correct, and unambiguous. Any typographical or grammatical errors should be corrected at revision, so please note any specific errors here.

Reviewer #1: (No Response)

Reviewer #3: Yes

________________________________________

6. Review Comments to the Author

Please use the space provided to explain your answers to the questions above. You may also include additional comments for the author, including concerns about dual publication, research ethics, or publication ethics. (Please upload your review as an attachment if it exceeds 20,000 characters)

Reviewer #1: (No Response)

Reviewer #3: Based on my review; The authors have provided the requested information by the reviewers and I would like to suggest this article is accepted. But, the authors needs to improve algorithm representations for the Algorithm 1 and 2. It will make the readers easy to know the algorithm flow.

________________________________________

7. PLOS authors have the option to publish the peer review history of their article (what does this mean?). If published, this will include your full peer review and any attached files.

If you choose “no”, your identity will remain anonymous but your review may still be made public.

Do you want your identity to be public for this peer review? For information about this choice, including consent withdrawal, please see our Privacy Policy.

Reviewer #1: No

Reviewer #3: No

________________________________________

**Referee Report**

**Journal:** PLOS ONE

**Title:** Innovative deep matching algorithm for stock portfolio selection using deep stock profiles

**Manuscript Number:** PONE-D-20-15612R1

**Date:** 2020/08/29

**Summary**

I appreciate the authors’ efforts and agree that the authors have done a good job in the previous round. There are only a few minor issues left. Specific comments are outlined below and I hope my comments can be useful for the authors to enhance this paper.

**Major Comments**

1. In the revised version, the abstract is much longer than the conclusions. There is no need to mention too many details in the abstract section. It would be better to swap the lengths of the two sections and summarize economic intuitions for the main results in the conclusions.

2. The roles of evaluation metrics of trading strategies in stock portfolio selection is not clearly explained. For example, Sharpe ratio is used to evaluate risk-adjusted performance, defined as the excess return divided by the standard deviation for a given trading strategy. Information ratio is used to measure the excess return of a trading strategy per unit excess risk, formulated as the excess return of a trading strategy divided by the annualized standard deviation of the daily return difference between a trading strategy and the benchmark. I would recommend the authors to explain how these two similar but different variables and other metrics affect stock portfolio selection.

Response: Thanks for the professional comments and suggestions. We have revised the manuscript and the changes made were highlighted in a marked-up copy of our manuscript in this way: added text is blue and set in sans-serif, and a red footnote is created for each discarded piece of text.

1. We have improved algorithm representations for the Algorithm 1 **in Page 6** and the Algorithm 2 **in Page 8**.
2. We have shortened the abstract section and removed some details **in Page 1**. Appropriate extensions have been made and economic intuitions for the main results were summarized in the conclusion section **in Page 17 line 680 to 699.**
3. We have explained the roles of evaluation metrics of trading strategies in stock portfolio selection and how these metrics affect stock portfolio selection **in Page 13 line 471 to 486.**
