# Supplementary material for: Innovative deep matching algorithm for stock portfolio selection using deep stock profiles
Source: PLoS One. 2020 Nov 4;15(11):e0241573. doi: 10.1371/journal.pone.0241573 (PMC7641377; doi:10.1371/journal.pone.0241573)
Supplement: Attachment — Submitted filename: 04 Referee Report PONE-D-20-15612R1.pdf [file pone.0241573.s017.pdf]

Referee Report

Journal: PLOS ONE
Title: Innovative deep matching algorithm for stock portfolio selection using deep stock profiles
Manuscript Number: PONE-D-20-15612R1
Date: 2020/08/29

Summary

I appreciate the authors' efforts and agree that the authors have done a good job in the previous round. There are only a few minor issues left. Specific comments are outlined below and I hope my comments can be useful for the authors to enhance this paper.

Major Comments

1. In the revised version, the abstract is much longer than the conclusions. There is no need to mention too many details in the abstract section. It would be better to swap the lengths of the two sections and summarize economic intuitions for the main results in the conclusions.

2. The roles of evaluation metrics of trading strategies in stock portfolio selection is not clearly explained. For example, Sharpe ratio is used to evaluate risk-adjusted performance, defined as the excess return divided by the standard deviation for a given trading strategy. Information ratio is used to measure the excess return of a trading strategy per unit excess risk, formulated as the excess return of a trading strategy divided by the annualized standard deviation of the daily return difference between a trading strategy and the benchmark. I would recommend the authors to explain how these two similar but different variables and other metrics affect stock portfolio selection.
